# Flagellin lysine methyltransferase FliB catalyzes a [4Fe-4S] mediated methyl transfer reaction

**DOI:** 10.1371/journal.ppat.1010052

**Published:** 2021-11-17

**Authors:** Chu Wang, Christian Nehls, Dirk Baabe, Olaf Burghaus, Robert Hurwitz, Thomas Gutsmann, Martin Bröring, Michael Kolbe

**Affiliations:** 1 Department of Structural Infection Biology, Center for Structural Systems Biology (CSSB) & Helmholtz-Center for Infection Research (HZI), Hamburg, Germany; 2 Division of Biophysics, Research Center Borstel, Leibniz Lung Center, Borstel, Germany; 3 Institut für Anorganische und Analytische Chemie, Technische Universität Braunschweig, Braunschweig, Germany; 4 Department of Chemistry, Philipps University of Marburg, Marburg, Germany; 5 Protein Purification Core Facility, Max Planck Institute for Infection Biology, Berlin, Germany; 6 MIN-Faculty University Hamburg, Hamburg, Germany; University of California Davis School of Medicine, UNITED STATES

## Abstract

The methyltransferase FliB posttranslationally modifies surface-exposed ɛ-N-lysine residues of flagellin, the protomer of the flagellar filament in *Salmonella enterica (S*. *enterica)*. Flagellin methylation, reported originally in 1959, was recently shown to enhance host cell adhesion and invasion by increasing the flagellar hydrophobicity. The role of FliB in this process, however, remained enigmatic. In this study, we investigated the properties and mechanisms of FliB from *S*. *enterica in vivo* and *in vitro*. We show that FliB is an S-adenosylmethionine (SAM) dependent methyltransferase, forming a membrane associated oligomer that modifies flagellin in the bacterial cytosol. Using X-band electron paramagnetic resonance (EPR) spectroscopy, zero-field ^57^Fe Mössbauer spectroscopy, methylation assays and chromatography coupled mass spectrometry (MS) analysis, we further found that FliB contains an oxygen sensitive [4Fe-4S] cluster that is essential for the methyl transfer reaction and might mediate a radical mechanism. Our data indicate that the [4Fe-4S] cluster is coordinated by a cysteine rich motif in FliB that is highly conserved among multiple genera of the Enterobacteriaceae family.

## Author summary

The bacterial flagella are tail-like appendages that play important roles in motility and host cell infection. In *Salmonella*, the surface of the flagellar filaments is heavily modified by methylation on their lysine residues, contributing to efficient gut colonization and successful invasion of the host. However, little is known about the properties of the methylase FliB, that catalyzes flagellar methylation. In this study, we isolated and characterized FliB of *S*. *enterica* for the first time. We show that FliB forms oligomers that localize in close proximity to the bacterial cell membrane. The FliB methyltransferase coordinates a [4Fe-4S] cluster at its active site by several cysteine residues that were highly conserved in many entero-pathogens. We further found that FliB modifies flagellar subunits using an iron-sulfur cluster dependent mechanism and requires a reducing environment. Our work sets a stage for understanding the roles of oxygen sensitive iron-sulfur cluster in flagellar modification and entero-bacterial pathogenesis.

## Introduction

Protein methylation plays a key role in physiological function and signaling pathway modulation. The first protein methylation was reported for lysine residues in the flagellin of *S*. *enterica* in 1959 by Ambler and Rees [1]. The gene *fliB* located between sigma factor gene *fliA* and flagellin gene *fliC* in chromosome was further identified to be responsible for the flagellin methylation [2–4].

Flagellin is the protomer (subunit) of the bacterial flagellar filament, which is a tubular supercoiled polymer assembled by ~20,000 flagellin subunits. The flagella protrude from the bacterial cell body and propel the bacterium for targeted movements. Besides locomotion, flagella also play important roles in host cell surface adhesion, colonization, biofilm formation and host inflammatory activation during infection [5–8]. The structure of the flagellin monomer is organized into four connected domains: the highly conserved D0 and D1 domains at both the N- and C-terminus forming the inner core of the filament structure and the variable D2 and D3 intervening domains forming the outer surface of the filament [9–11] (S1 Fig).

The flagellin ɛ-N-lysine methyltransferase gene *fliB* was found universally and vertically maintained throughout several monophylogenetic lineages in Enterobacteriaceae [12]. The encoded enzyme FliB functions for posttranslational modification on many of the lysine residues in flagellin to N-methyl-lysine. It has been reported that almost all the lysine residues at flagellin out-facing D2 and D3 domains are methylated, while lysine residues at flagellar inner core (D0 and D1 domains) are not modified [13–15] (S1 Fig). Although the flagellin methylation has been discovered since middle of last century, the enzymatic properties of FliB and the functions of the flagellin methylation remained unclear. The latter might be explained by the observation that the loss of *fliB* has no impact on bacterial swimming behavior [4]. Our recent study on the role of flagellin modification demonstrated that the posttranslational flagellin methylation promotes bacterial adhesion and host cell invasion by increasing the flagellar surface hydrophobicity [15]. While the importance of the flagellin methylation on cell invasion was unraveled, the methyltransferase mechanism of FliB is still poorly understood.

In this study, we purified the ɛ-N-methyl-lysine methyltransferase FliB of *S*. *enterica* serovar Typhimurium that showed for the first time *in vitro* enzymatic activity. By characterizing the biochemical and biophysical properties of FliB, we demonstrate that FliB forms a membrane associated homooligomer that modifies flagellin using a [4Fe-4S] and SAM dependent methyl transfer reaction.

## Results

### FliB functions in cytoplasm and localizes near cell membrane

The flagellin lysine methyltransferase FliB is conserved among multiple bacterial species of the Enterobacteriaceae family, particularly in pathogenic strains [12] (S2 Fig). Sequence-based predictions suggested that FliB might localize in the cytoplasm and consist mainly of helices adopting a stable fold [16–19].

**Fig 1:**
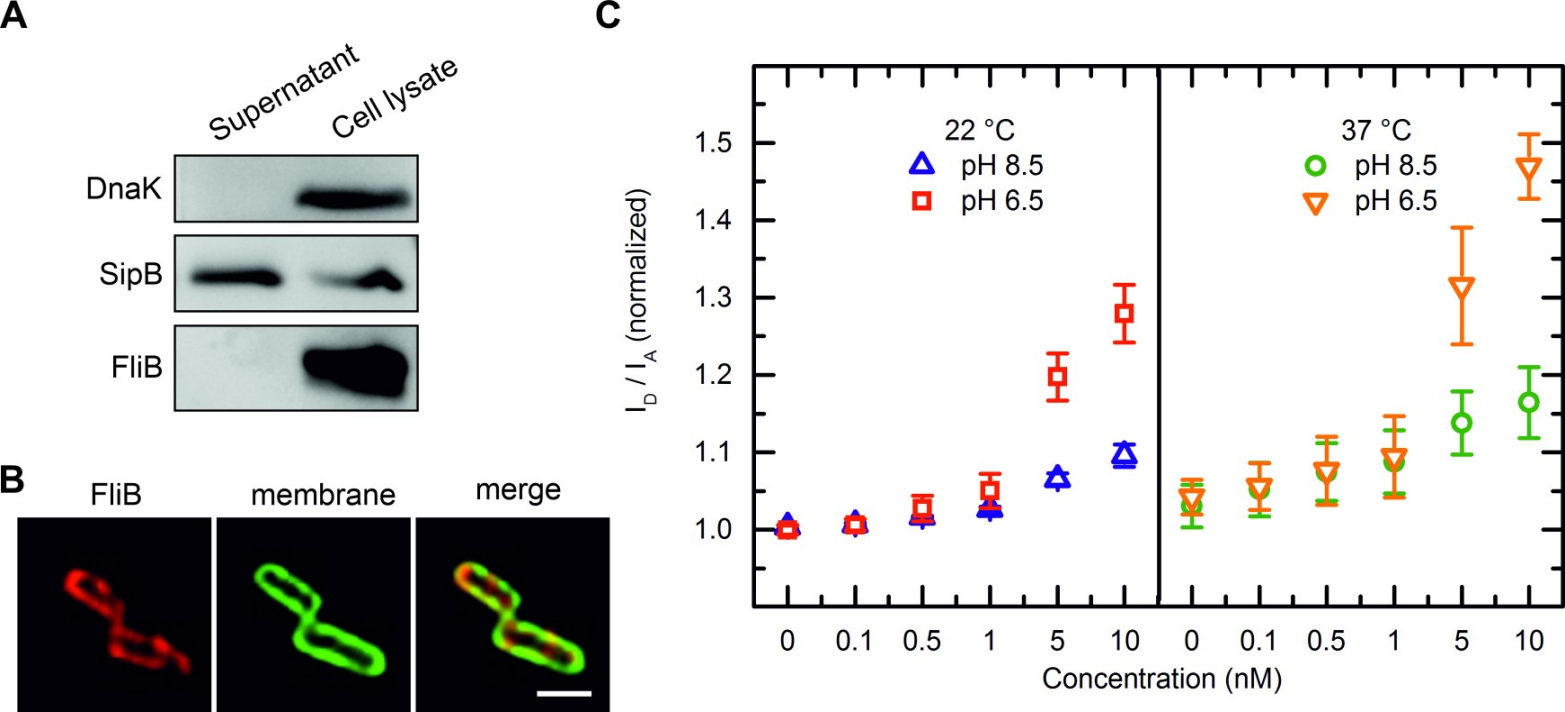
FliB localization. (**A**) Western blot analysis of proteins from *S*. *enterica* Typhimurium whole cell lysates and protein secreted into the growth culture. The protein DnaK served as cell lysis control and the secreted protein SipB as loading control (positive control). (**B**) High magnification micrographs showing the FliB localization in *Salmonella* cells. The rod-shaped *Salmonella* cells were visualized, FliB was detected by using an anti-strep primary antibody in combination with a secondary antibody conjugated with the Alexa 647 (red) and the cell membrane was labelled with *FAST* DiO lipid dye (green) (see also S4 Fig). The scale bar corresponds to 1 μm. (**C**) FRET spectroscopy of concentration dependent ftFliB association with liposomes. Titration of ftFliB (up to 10 nM) to a PE:PG (1:1 [m:m]) liposome population (10 μM lipid concentration) that was double labelled with NBD-PE as FRET donor and rh-PE as FRET acceptor lead to a reduced FRET efficacy. This can be derived from the fluorescence intensity ratio donor to acceptor, which is plotted against the concentration. At 22°C, the ftFliB interaction with lipid matrix was stronger at pH 6.5 than at pH 8.5; the same relationship applied at 37°C, with more pronounced effects at both pH. Error bars indicate standard deviations of 4 or 5 independent measurements.

The fact that FliB methylates flagellin on almost all the lysine residues at its surface exposed domains [13–15] (S3 Fig) raises a question, whether the enzyme modifies folded flagellin subunit or is being secreted and functions after flagellar extracellular assembly? To address this question, we generated a *Salmonella* strain carrying strep-tag vector with *fliB* gene originated from *S*. *enterica* Typhimurium. We conducted protein secretion assays and found no FliB released into the culture supernatant while the protein could be detected in the cell lysate (Fig 1A). We further analyzed the FliB localization using immunofluorescence microscopy. Imaging experiments showed that FliB locates in the bacterial cytoplasm in close vicinity to the inner membrane where it forms distinguishable clusters (Figs 1B and S4).

### Purification and characterization of FliB

We made several attempts to overproduce and purify FliB from both *E*. *coli* and *S*. *enterica* strains. However, only little amounts of FliB was observed in the soluble fraction and efforts to purify the meager amounts resulted in inhomogeneous and instable enzyme that precipitated when concentrated higher than 0.5 mg/mL. In order to obtain soluble methyltransferase, we screened the effects of several solubility tags fused to FliB. Eventually we acquired soluble and stable protein using a genetic construct that produces FliB fused to the C-terminal of truncated FleB protein (FleBt), a *Yersinia enterocolitica* flagellin protein lacking D0 domain (S5A Fig). The N-terminal his-tagged FleBt tag has been used previously to obtain sufficient amounts of soluble and folded proteins, which are notoriously difficult to produce [20]. It is also worth to mention that FleB shares a conserved D1 domain with the substrate flagellin of FliB (S3 Fig) and therefore might also contribute to stabilize FliB in the fused protein. Coomassie blue stained SDS-PAGE and Western blotting confirmed that the FleBt-FliB (ftFliB, ~76 kDa) fusion protein can be produced and purified from *E*. *coli* cells in good amount and with high purity (S5B Fig). Next, we evaluated the stability of ftFliB using nano differential scanning fluorimetry (nanoDSF). The thermal denaturation curve of ftFliB showed an onset unfolding temperature of (28.2 ± 1.0)°C and a melting temperature (Tm) of (33.8 ± 0.7)°C (S6A Fig), while the scattering curve showed that ftFliB started to aggregate at (57.1 ± 1.3)°C (S6B Fig). These results indicate that ftFliB is stably folded at room temperature or below. Similar Tm was observed in other bacterial methyltransferases [21,22]. As the cleavage of FleBt tag lead to FliB precipitation, we decided to work with the fusion proteins if not stated otherwise in all the following experiments.

To characterize the membrane association of FliB more closely, we used a Förster Resonance Energy Transfer Spectroscopy (FRET) assay to monitor the interaction between the purified protein and phospholipid vesicles. At a suitable concentration of lipid-coupled donor and acceptor fluorophores, the protein-membrane interaction can be detected from an increase in the mean donor-acceptor distance, which leads to a reduction in FRET efficiency [23]. In titration experiments of vesicles composed of phosphatidylethanolamine (PE) and phosphatidylglycerol (PG), serving as a simple model for *Salmonella* membranes, ftFliB caused a clearly reduced FRET efficacy at protein concentrations as low as 0.5 nM (Fig 1C). This corresponds to a lipid:protein ratio of 20000:1 or a weight ratio of approximately 200:1. The assays also indicated that ftFliB-membrane association were stronger at 37°C compared to 22°C and considerably stronger at pH 6.5 compared to pH 8.5. Controls with buffer or with the solubility tag FleBt showed no or only negligible effect (S7 Fig), confirming that the fusion protein membrane affinity was derived from the methyltransferase. Corroborating with the secretion assay and microscopy results, we show that FliB is a non-secreting cytosolic protein and associates with the cytoplasmic membrane.

### FliB contains a [4Fe-4S] cluster

**Fig 2:**
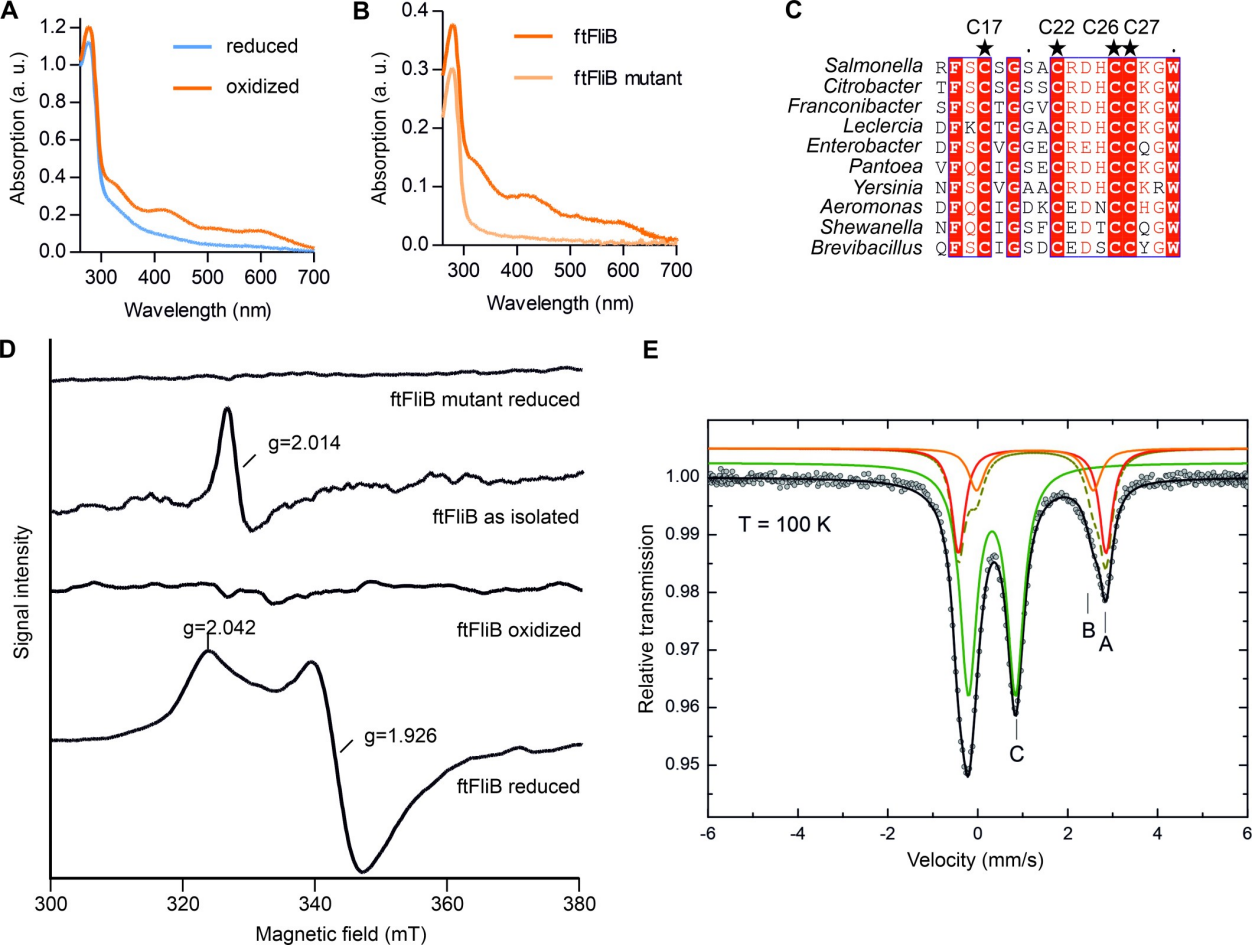
Spectroscopic characterization of FliB [4Fe-4S] cluster. (**A**) UV-vis absorption spectra of oxidized (as purified) and dithionite reduced ftFliB (~160 μM, reconstituted protein). (**B**) UV-vis absorption spectra of reconstituted wild type (ftFliB) and ftFliB ^C17A C22A C26A C27A^ mutant (ftFliB mutant, ~50 μM). (**C**) Alignments of FliB N-terminal amino acid sequences from multiple genera (S2 Fig). Conserved cysteines are marked with stars. (**D**) X-band EPR spectra of ftFliB at T = 10 K treated with dithionite under strictly anaerobic condition (ftFliB reduced); ftFliB treated without dithionite and exposed to oxygen for long (ftFliB oxidized); ftFliB isolated under reducing condition but was shortly exposed to oxygen (ftFliB as isolated); and ftFliB mutant treated with dithionite anaerobically (ftFliB mutant reduced). (**E**) Zero-field ^57^Fe Mössbauer spectrum of anaerobic reconstituted ^57^Fe enriched ftFliB, recorded at T = 100 K. Symbols: Experimental data. Lines: Fit with doublets of Lorentzian lines. The coloured solid lines illustrate the corresponding sub-spectra (A, B and C) of the fit, while the black line represents the superposition of these components A, B and C. The parameters of the fit are summarized in Table 1.

Purified ftFliB showed a brownish color with two absorption peaks at 315 nm and 410 nm in UV-vis absorption spectra (Figs S8A and 2A), indicating the presence of [4Fe-4S] clusters [24]. In contrast, the purified FleBt solubility tag at similar concentration was colorless, indicating the iron-sulfur binding by FliB. Multiple sequence alignment of FliB with orthologous proteins indicated the presence of a conserved cysteine rich cluster featured as CX_4_CX_3_CC at the N-terminal of the protein sequences (Figs 2C and S2). Similar cysteine motifs (CX_2_CX_2_CC, PF03692) were attributed with chelating effects on zinc or iron ions [25]. We next analyzed the purified ftFliB sample using X-ray fluorescence (XRF) and X-ray absorption near edge structure (XANES) spectroscopy to identify the metal binding. The obtained spectra confirmed the iron presence in ftFliB and suggested the absence of other metals bound to the methyltransferase (S8D and S8E Fig).

Consequently, ftFliB was chemically reconstituted under strict anaerobic conditions, yielding a dark brown protein solution (S8B Fig). The absorption peaks at 315 nm and 410 nm disappeared when the reconstituted protein was further reduced with 10 mM sodium dithionite (Figs 2A and S8C), which is typical for iron-sulfur proteins [24]. A determination of the iron and sulfur content of the reconstituted protein revealed that (4.14 ± 0.37) mol Fe and (4.62 ± 0.49) mol S were bound per mol protein, indicating the presence of one [4Fe-4S] cluster in FliB. To further test the role of CX_4_CX_3_CC motif in iron-sulfur binding, we generated a quadruple-alanine mutant of the cysteine residues (ftFliB ^C17A C22A C26A C27A^). The purified mutant protein was colorless and other than in the wild-type protein no peaks could be detected in UV-vis absorption spectra at 315 and 410 nm (Fig 2B).

Next, we used X-band electron paramagnetic resonance (EPR) spectroscopy to analyze the type and the oxidization states of the iron-sulfur cluster in ftFliB and its cysteine mutant. At T = 10 K, the isolated ftFliB (without addition of dithionite) exhibited an EPR signal at g = 2.014, which is typical for [3Fe-4S]^+^ [26]. The strictly reduced ftFliB with addition of dithionite under anaerobic condition showed an EPR signal with resolved g-tensor components (g|| = 2.042, g⊥ = 1.926) characteristic for [4Fe-4S]^+^ cluster [26] (Fig 2D). In comparison, the oxidized ftFliB by long exposure to air became EPR inactive, as an indication for [4Fe-4S]^2+^ clusters (Fig 2D). Note, the above-mentioned cysteine mutant ftFliB ^C17A C22A C26A C27A^ did not show an iron-sulfur EPR signal in both reduced and oxidized states, corroborating the role of the CX_4_CX_3_CC motif in coordination of a [4Fe-4S] cluster. Zero-field ^57^Fe Mössbauer spectroscopy further confirmed the presence of [4Fe-4S] cluster using an anaerobic reconstituted ^57^Fe enriched ftFliB sample. The corresponding Mössbauer spectrum at T = 100 K is dominated by a doublet (Fig 2E, green line, C, 64% total intensity) with isomer shift of *δ* = 0.437(2) mm s^-1^ and quadrupole splitting of Δ*E_Q_* = 1.052(4) mm s^-1^ (Table 1), which can be assigned to a [4Fe-4S]^2+^ cluster [27]. The weaker absorption peaks (Fig 2E, dark yellow, broken line, 36% total intensity) describe the superposition of the two sub-spectra (Fig 2E, red and orange lines, A and B) associated with adventitiously bound high-spin Fe (II) in the protein, presumably introduced during the reconstitution procedure. These results further indicate that the primary cluster form after dithionite reduction is [4Fe-4S]^2+^. The dithionite reduced [4Fe-4S]^+^ state, which was clearly observed by EPR measurement, might only exist transiently. Similar spectral features were observed previously for radical SAM proteins PhDph2 [28], PFL-AE [29] and HemW [30], all containing [4Fe-4S] clusters.

In summary, all the spectroscopic data consistently suggest that FliB contains a [4Fe-4S] cluster, which is able to transit between [3Fe-4S]^+^, [4Fe-4S]^+^ and [4Fe-4S]^2+^ states under different conditions.

**Table 1:** Mössbauer spectrum parameters of anaerobic reconstituted ^57^Fe enriched ftFliB.

T (K)	*δ* ^[a]^ (mm s^-1^)	Δ*E_Q_* (mm s^-1^)	Γ_FWHM_ (mm s^-1^)	AREA (%)	cf., Fig 2E
100	0.437(2)	1.052(4)	0.444(4)	64.0%	C
	1.395(7)	2.59(3)	0.43(3)	12.2%	B
	1.333(2)	3.29(1)	0.35 (1)	23.7%	A

Summary of Mössbauer parameters determined for ftFliB as in Fig 2E by a fit with three doublets of Lorentzian lines (A, B and C), with isomer shift *δ*, quadrupole splitting Δ*E_Q_* and Lorentzian line width Γ_FWHM_ (full width at half maximum). AREA quotes the relative (integral) intensities of an individual doublet of Lorentzian lines. [a]: Isomer shifts were specified relative to metallic iron at room temperature but were not corrected in terms of the second-order Doppler shift.

### Flagellin methylation activity of FliB

**Fig 3:**
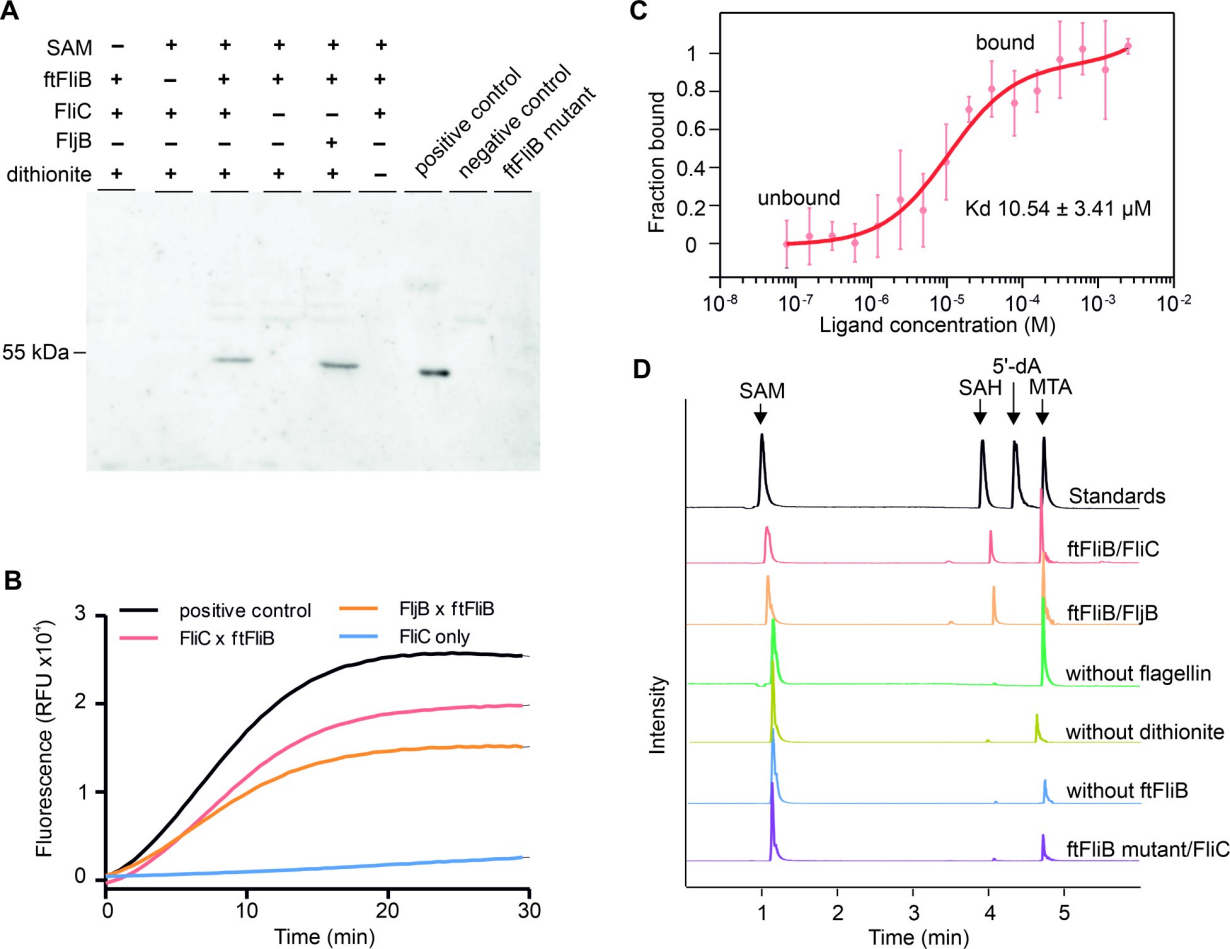
*In vitro* FliB methylation activity. (**A**) Activity assay using an anti-methylated Lysine antibody. Methylated flagella sheared from wild type *S*. *enterica* were used as positive control; unmethylated flagella sheared from *S*. *enterica* FliB knockout mutant were used as negative control. Reaction with ftFliB ^C17A C22A C26A C27A^ (ftFliB mutant) was conducted in a mixture of SAM, FliC and dithionite. (**B**) Continuous fluorescent methyltransferase assay, SAH was used as a positive control and buffer alone was used as background control. Methylase ftFliB was mixed with either FliC or FljB under reducing environment, and the generation of SAH was measured using enzyme coupled fluorescence assay with an excitation/ emission wavelength of 535/590 nm. (**C**) Ligand binding affinity test of ftFliB and SAM. Binding of methylation ligand SAM to 1 μM ftFliB was analyzed with label-free MST. Data were shown as mean ± standard deviation (SD) calculated from three independent experiments. (**D**) HPLC-MS analysis of the methylation assay products. The methylation reactions were performed by mixing the methylase (ftFliB) and flagellin under reducing condition: reaction between ftFliB and FliC as ‘ftFliB/FliC’; reaction between ftFliB and FljB as ‘ftFliB/FljB’, control reaction lack of flagellin as ‘without flagellin’, control reaction under non-reducing condition as ‘without dithionite’, control reaction without the methylase as ‘without ftFliB’, reaction of ftFliB mutant with flagellin as ‘ftFliB mutant/FliC’. Extracted ion chromatograms (EIC) of SAM (m/z 399 [M + H]^+^), SAH (m/z 385 [M + H]^+^), 5’-dA (m/z 252 [M + H]^+^), MTA (m/z 298 [M + H]^+^) from each reaction were merged in one trace. The chromatograms intensities are normalized to the most abundant EIC.

In order to investigate the *in vitro* activity of ftFliB, we performed multiple methyltransferase assays by mixing purified *S*. *enterica* flagellins and ftFliB together with the methyl donor S- adenosylmethionine (SAM) as indicated in previous *in vivo* experiment [31]. In *S*. *enterica*, flagellin is alternatively expressed as protein FliC or FljB in a process known as phase variation [32]. In order to check the prevalence of the methylation reaction, we purified both phases of flagellins as substrates. Using an anti-methylated lysine antibody, we showed that flagellin, either FliC or FljB were methylated in the presence of ftFliB and SAM under reducing condition (Fig 3A). In the absence of SAM, enzyme (ftFliB), substrate (FliC or FljB), or dithionite the methylation could not be detected. The mutant ftFliB ^C17A C22A C26A C27A^ also ablated methylation, indicating the importance of the CX_4_CX_3_CC motif for the FliB activity (Fig 3A). Next, we continuously monitored the methyltransferase activity using a fluorescence-based *Methyltransferase Assay Kit* (SAMfluoro, G-Biosciences), recording the generation of S-adenosylhomocysteine (SAH) from SAM during the methylation reaction. We observed that SAH was immediately generated when ftFliB, flagellin and SAM were mixed, while no SAH was detected in the absence of ftFliB (Fig 3B). The methyltransferase activity was calculated as (110.5 ± 12.1) nmol/min fluorophore per mg protein based on a resorufin standard assay. We further conducted label free microscale thermophoresis (MST) to analyze the binding affinity of ftFliB to SAM (Fig 3C), resulting in an equilibrium dissociation constant (Kd) of (10.54 ± 3.41) μM. A ligand-induced fluorescence change was observed when increasing concentration of SAM was added to ftFliB, indicating structural changes of ftFliB upon binding with SAM.

The existence of an iron-sulfur cluster and the dependency of SAM for the enzymatic reaction suggested that FliB might use the cluster to generate radicals for the methylation reaction. For further exploration of the reaction mechanisms of FliB and the role of the [4Fe-4S] cluster, we used high-performance liquid chromatography-mass spectrometry (HPLC-MS) to characterize the methylation products. In the reaction containing SAM, ftFliB, dithionite and either FliC or FljB, the SAM molecules were converted to SAH and methylthioadenosine (MTA; Fig 3D). The situation is different in the absence of flagellin, where MTA was generated but almost no SAH could be detected. In control reactions excluding either the methyltransferase (ftFliB) or dithionite, only low concentrations of MTA and almost no SAH was observed, while most SAM molecules were left intact. These observations are consistent with the activity assay results shown in Fig 3A and 3B. Cleavage of the S-C (5’) bond of SAM did not occur in our reaction because the formation of 5’-deoxyadenosine (5’-dA), which is the most likely radical intermediate of the adenosyl moiety [24], was not detected. However, we observed an increase of MTA in the presence of ftFliB and dithionite, suggesting that FliB might catalyze the cleavage of the S-C (γ) bond of SAM, forming a 3-amino-3-carboxypropyl (ACP) radical and releases the remaining MTA. Similar observations in radical enzymes glycerol dehydratase [33] and phDph2 [28] were reported previously.

### FliB forms specific stable oligomer

**Fig 4:**
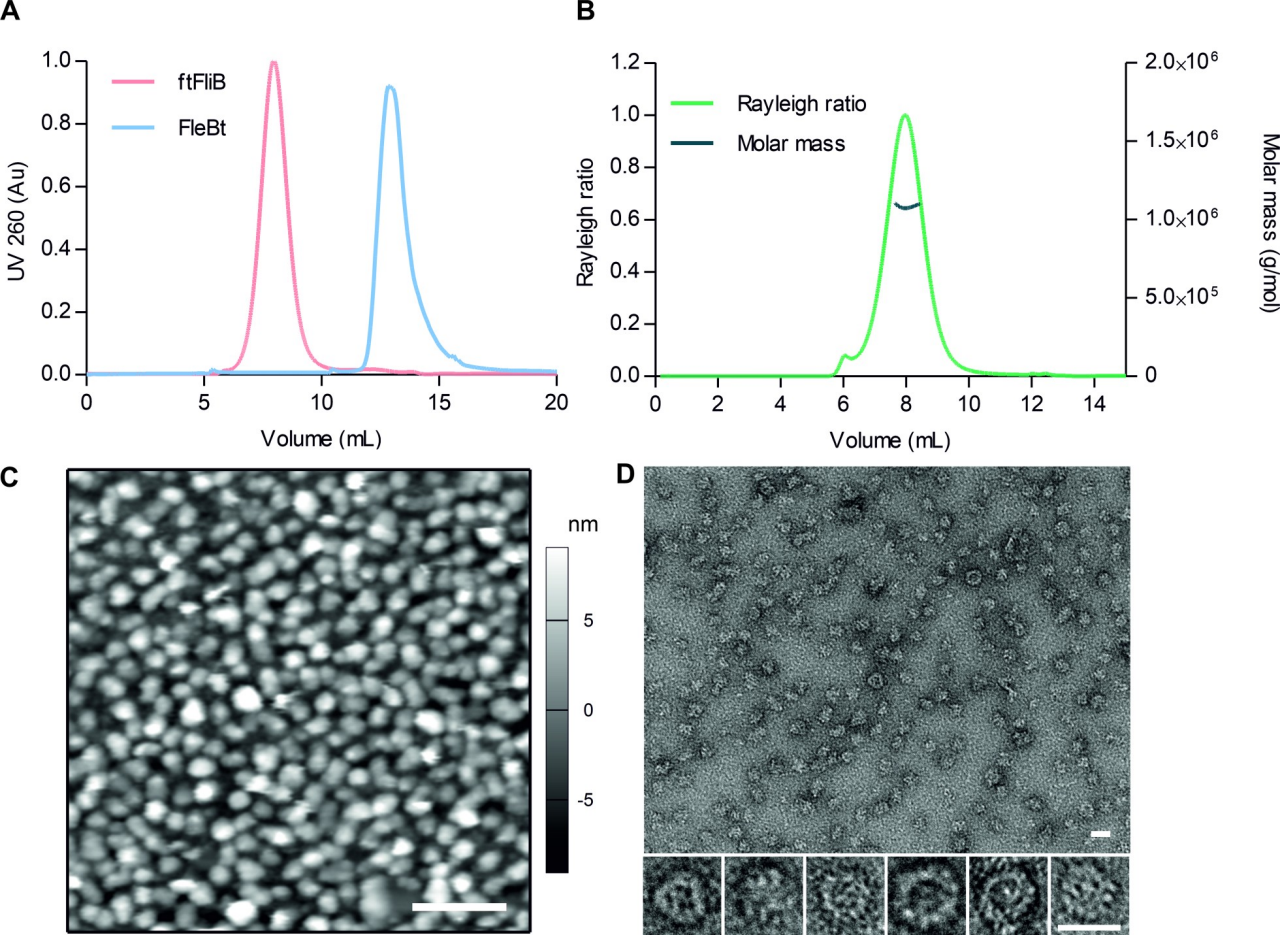
FliB forms a homo-oligomer. (**A**) Comparison of molecular size-exclusion chromatography of ftFliB and FleBt tag carried out on a TSK-GEL BioAssist G4SW_XL_ column. (**B**) SEC-MALS analysis of ftFliB. The Rayleigh ratio (light scattering, LS) profile (light green, left axis) shows one major peak. The molar mass trace (dark green, right axis) across the elution peak derived from RI/LS measurements corresponds to homogeneous oligomeric states of ftFliB. (**C**) 2D-AFM height profile picture of purified ftFliB oligomers in buffer that have settled on a flat MICA surface. The grayscale color profile ranges from -9 nm (black) to 9 nm (white). Scale bar is 100 nm. (**D**) TEM of purified ftFliB oligomers with particles of distinct globular shape enlarged below. Scale bars correspond to 25 nm.

The fusion protein ftFliB formed an oligomer and eluted at high molecular weight during size exclusion chromatography. In comparison to that, the solubility tag (FleBt) in the absence of FliB eluted as monomer (Fig 4A), suggesting that the protein oligomerization depends on the presence of FliB. In order to evaluate the oligomer state and the molecular mass of purified ftFliB, we carried out analytical gel-filtration chromatography coupled to multi-angle light-scattering (SEC-MALS), refractive index (RI) and UV_280 nm_ detectors (Fig 4B). Analysis of the scattering experiments indicated molecular masses of (1.104×10^6^ ± 0.431×10^3^) g/mol and (1.083×10^6^ ± 0.433×10^3^) g/mol for ftFliB based on RI and UV_280 nm_ measurements, respectively. These molecular masses correspond to approximately 14 subunits per oligomer on average.

The homogeneity and shape of the ftFliB oligomer were further assessed by atomic force microscopy (AFM) and transmission electron microscopy (TEM) (Fig 4C and 4D). Comparable particle sizes and the spherical shapes of the complex were observed in both measurements. The mean diameter of the oligomers was determined as (21.7 ± 2.7) nm by AFM measurements and (19.9 ± 1.6) nm by TEM measurements. The mean height of all image points allocated to the oligomers was (4.6 ± 0.5) nm and the maximum height of the oligomers was about 15 nm, estimated from the AFM results (Fig 4C). Taken together, the ftFliB forms a spherical complex with a molecular mass in the Megadalton range suggesting a tetradecameric assembly.

## Discussion

Iron-sulfur clusters play important roles in enteropathogenic bacteria by regulating their virulence profiles rapidly in response to the changing iron availability, oxygen tension and levels of reactive oxygen species in the host environment [34]. In this study, we identified the *S*. *enterica* virulence factor FliB, which promotes bacterial adherence to host cells [15], as a novel [4Fe-4S] cluster enzyme conserved in many Gram-negative pathogens. The ɛ-N-methyl-lysine methyltransferase FliB modulates flagellar surface properties by performing a methylation reaction that is subject to a CX_4_CX_3_CC motif bound [4Fe-4S], a methyl donor SAM and a reducing condition.

Lysine methylation reaction is usually mediated by nucleophilic SN2 mechanism and heterolytic cleavage of SAM using methylase that contain conserved sequence motifs for SET domains or Rossmann folds [35–37]. Bioinformatics analysis of FliB however, did not reveal the presence of any characterized protein domain or sequence similarity with known protein structures. On the other side, with the surprisingly found [4Fe-4S] cluster and the reaction products, FliB resembles to radical SAM enzymes, which reductive cleave SAM through a cysteine coordinated [4Fe−4S] cluster [24]. The existence of the [3Fe-4S]^+^ state further indicates that one of the irons in the [4Fe−4S] cluster might be unique and subject to ligand binding upon reduction [29]. Interestingly, we noticed that FliB harbors 8 highly conserved cysteine residues (S1A Fig) but only coordinates one [4Fe-4S] cluster in the enzyme. The remaining conserved cysteines might play roles in facilitating the methyl transfer as reported for radical SAM methyltransferases RlmN and Cfr previously [38,39]. Moreover, the sensitivity of FliB to oxygen also rationalize the difficulties in previously reported *in vitro* methylation experiments [31]. In order to understand the ligand-protein interaction mechanisms in detail, additional structural and spectroscopic studies would be required.

Indeed, the flagellin methylation pattern is distinct from other known protein methylation reactions that are mostly specific and catalyzed by sequence-orientated methyltransferases. However, FliB methylates almost all the lysine residues at flagellar filament surface exposed domains (S1B Fig) and no clear amino acid sequence consensus was identified flanking the methyllysines [15]. It seems that the FliB methylation displays a strong correlation between flagellin structure and the sites of modification- supporting this, Yoshioka et al. reported that flagellin mutants lacking part of the surface domain (D3) showed shifted methyllysine distributions due to altered surface exposures of flagellin tertiary structure [40]. Noteworthy, the structure-dependent methylation at substrate surface accessible sites was also observed in Archaea [41].

**Fig 5:**
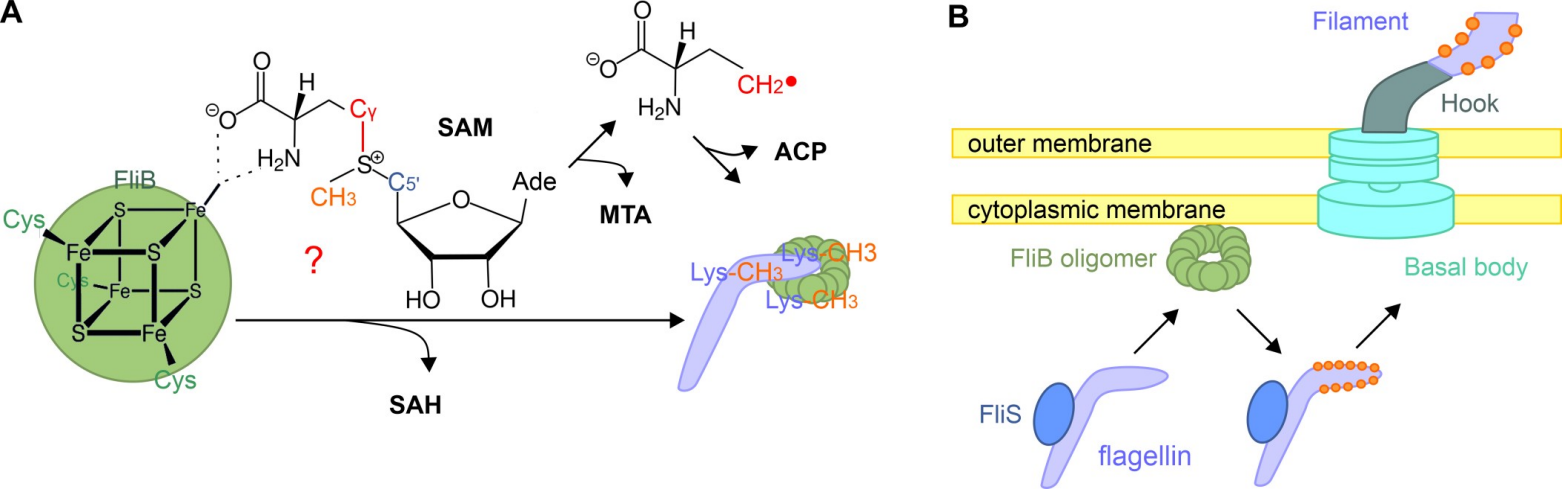
The proposed reaction mechanism for FliB. (**A**) The mechanism assumes two roles of SAM binding to [4Fe-4S] in FliB: One SAM is converted into an ACP radical and facilitates the methyl transfer from the other SAM molecule to the flagellin substrate. (**B**) The postulated process illustrates that the flagellin subunit bound by its chaperon FliS is delivered to the FliB oligomer near the cytoplasmic membrane. The *Salmonella* FliB complex methylates accessible lysine residues in flagellin domains D2 and D3 before protomer secretion and flagellar assembly.

Because of the unique flagellin methyllysine pattern, Parish et al. proposed that methylation may occur after polymerization of the flagellin [13]. However, our data demonstrate that FliB is not secreted but rather forms an oligomer and locates near cell membrane in clusters. Taken into account that flagellin polymerization is suppressed in the cytosol by forming a complex with chaperon FliS, we propose that FliB targets and methylates flagellin subunits exclusively in the cytosol before flagellin secretion by flagellar apparatus (Fig 5B). Our proposal is in agreement with the previous observations from Tronick et al., who detected the monomeric methylated flagellin in the cell lysate and demonstrated that the flagellin methylation happens prior to its polymerization [31].

Collectively, we postulate a mechanism that prior to flagellin secretion from cytoplasm, flagellin is delivered in complex with its chaperon FliS to the FliB oligomer that is located near the bacterial inner membrane; the ɛ-N-lysine methyltransferase FliB recognizes the D2 and D3 domains of flagellin and methylates the surface lysines using a [4Fe-4S] mediated methyl transfer reaction (Fig 5).

## Methods

### Protein secretion assay

The *fliB* gene amplified from *S*. *enterica* serovar Typhimurium strain SL1344 was cloned into vector pASK-IBA3C (IBA GmbH) with a strep-tag at the C-terminal of *fliB* (*fliB*-strep). The *S*. *enterica* carrying this construct was grown at 37°C to an OD_600_ of ~0.4 in lysogenic broth (LB) media supplemented with 6 μg/mL chloramphenicol. The *fliB*-strep expression was induced at 25°C by addition of 20 ng/mL AHT for 3 h.

Secreted proteins were precipitated from filtered bacterial culture supernatants by addition of 15% ice-cold trichloroacetic acid (TCA) and centrifugation at 16,000 g for 30 min. Pellets were washed with ice-cold acetone, air-dried and re-suspended in buffer (100 mM Tris, 150 mM NaCl pH 8.0). Samples were loaded onto SDS-PAGE gels and analyzed by Coomassie staining and Western blot. Mouse *Strep*-tag monoclonal antibody (Qiagen) was applied for detection of FliB in Western blotting analysis. Rabbit anti-SipB polyclonal antibodies and anti-DnaK antibodies (StressGen Biotechnologies) were used as loading control and lysis control, respectively. HRP conjugated secondary antibodies and ECL Western blotting substrates were used for protein detection.

### Immunolabeling of protein and confocal microscopy

The *S*. *enterica* carrying *fliB*-strep was grown at 37°C to an OD_600_ of ~0.4 in LB media supplemented with 6 μg/mL chloramphenicol. The production of FliB-strep was induced by addition of 20 ng/mL AHT at 25°C for 3 h.

The bacterial cells were collected and prepared as previously described for microscopy [42]. In brief, the cells were fixed with 2.8% formaldehyde and 0.04% glutaraldehyde for 15 min at room temperature, permeabilized with 0.1% Triton and 100 μg/mL lysozyme for 45 min each. The cells were then blocked with 10% BSA in PBS containing 50 mM ammonium chloride. To detect the strep-tagged FliB, mouse *Strep*MAB-Classic antibody (iba, 10 μg/mL) and goat anti-mouse IgG, Alexa Fluor 647 (Invitrogen, 5 μg/mL) were used for immunolabeling. *FAST* DiO (5 μg/mL, Invitrogen) was added for cell membrane staining. Control samples were treated with goat anti-mouse IgG, Alexa Fluor 647 and *FAST* DiO only.

For localization imaging, cells were immobilized with mowiol mounting medium and imaged with a Leica TCS SP8 confocal microscope (Leica Microsystems) with a 63x 1.4 oil immersion objective and HyD detectors. The high-resolution images were acquired using the HyVolution mode of the Leica LASX software and Huygens software for deconvolution. Images were further processed using Fiji [43].

### Cloning, expression and purification of ftFliB

The *fliB* gene amplified from *S*. *enterica* serovar Typhimurium strain SL1344 was cloned into vector pET28a (Novagen) harboring an N-terminal His_6_- and the *Yersinia*-derived truncated flagellin FleB (FleBt, residues 54 to 332) as a solubility tag. *E*. *coli* BL21 (DE3) containing this construct was grown at 37°C to an OD_600_ of ~0.3 in LB containing 50 μg/mL Kanamycin before supplemented with trace metal mix (50 μM FeCl_3_, 20 μM CaCl_2_, 10 μM MnCl_2_, 10 μM ZnSO_4_, 2 μM CoCl_2_, 2 μM CuCl_2_, and 2 μM NiCl_2_) [44] and 200 μM L-Cysteine. When the culture was grown till OD_600_ of ~0.5, the protein production was induced with 0.25 mM IPTG, followed by 18 h expression at 17°C and harvested by centrifugation.

All purification steps were performed at 4°C. The cell pellets were re-suspended in buffer A (50 mM Tris-HCl, 150 mM NaCl, 10% glycerol, pH 8.5) supplemented with 2 mg/mL lysozyme, protease inhibitor cocktail (Roche), 10 μg/ml DNase I and 10 mM imidazole. Cell lysis was achieved by sonication (Bandelin sonopuls), and debris was removed by centrifugation at 48,000 g for 45 min.

The protein was immobilized on HisTrap HP columns (GE Healthcare), washed with buffer A containing 10 mM Imidazole, 3 mM ATP and 10 mM MgCl_2_ in order to remove unbound proteins and chaperone contamination and eluted with buffer A containing 500 mM imidazole. Affinity-purified proteins were polished by size exclusion chromatography (SEC) on a Superose 6 (GE Healthcare) or a TSK-GEL BioAssist G4SW_XL_ column (TOSOH BIOSCIENCE) equilibrated with buffer A, with ftFliB having been desalted against the same buffer using PD-10 column (GE healthcare) before the SEC. Peak fractions containing ftFliB were pooled and analyzed by SDS-PAGE followed by Coomassie-staining or Western blot. The ftFliB ^C17A C22A C26A C27A^ mutant and the FleBt solubility tag were expressed and purified in the same manner.

### Reconstitution of iron-sulfur clusters in ftFliB

After affinity purification, ftFliB was desalted against buffer A using PD-10 column and concentrated to 100–200 μM using Amicon filter units (Millipore). The protein was then reconstituted under anaerobic conditions on ice: 60 equiv. of DTT were added to the protein and incubated for 1 h; 6 equiv. of ammonium iron citrate were slowly added and incubated for 5 min; 6 equiv. of lithium sulfide were added and incubated overnight. The dark brown solution was further desalted with PD-10 column into buffer A to remove unbound substances.

### FRET measurements

Small unilamellar vesicle (SUVs) were prepared as described previously [45] by ultrasonic sound from *E*. *coli* PE and PG (1:1 molar ratio, Avanti Polar Lipids) to a total lipid concentration of 1 mM in pH 8.5 buffer or pH 6.5 buffer (50 mM Tris, 200 mM NaCl). Labelling of liposomes for FRET spectroscopy was achieved by addition of 0.5% NBD-PE (Life Technologies) as donor dye and 0.5% rho-PE (Life Technologies) as acceptor dye.

Using a Fluorolog 3 fluorescence spectrometer (HORIBA Ltd.), a FRET spectroscopy probe dilution assay [23] was used to investigate the interaction ftFliB has with membranes consisting of bacterial lipids. Five times 20 μl protein solution were added to 1900 μL 10 μM PE:PG liposomes. Thereby the protein concentrations 0.1 nM, 0.5 nM, 1 nM, 5 nM and 10 nM were obtained. The latter concentration corresponds to a lipid-protein ratio of 1000:1.

A possible intercalation of ftFliB units between the lipid molecules of the liposomes leads to an increase in the mean distance between donor and acceptor dyes. By excitation of the donor dyes at 470 nm, this FRET effect was recorded as an increase of the ratio between donor intensity I_D_ at 531 nm and acceptor intensity I_A_ at 593 nm as a function of time. Experiments were performed to analyze the influence of buffer pH (8.5 and 6.5) and the influence of temperature (22°C and 37°C) on protein intercalation. For both pH and temperature conditions, titration of buffer and titration of FleBt were used as controls. All experiments were performed at least 4 times.

### UV-vis spectroscopy

UV-vis analysis of purified ftFliB and its mutant was conducted on NanoDrop 2000 spectrophotometer (Thermo Fisher Scientific). To obtain reduced protein 10 mM sodium dithionite was added to the protein solution.

### Iron and sulfur content measurement

The quantification of iron and sulfur content was measured as described previously [46]. In brief, for iron content measurement, 100 μL of purified ftFliB sample was mixed with 100 μL 1% HCl and incubated for 10 min at 80°C to denature the protein. Afterwards 500 μL 7.5% ammonium acetate (w/v), 100 μL 4% ascorbic acid, 100 μL 2.5% SDS and 100 μL 1.5% ferene were added and centrifuged at 9000 rpm for 5 min, resulting in a blue solution with an absorption wavelength of 593 nm. For sulfur content measurement, 200 μL of purified ftFliB sample was mixed with 600 μL 1% zinc acetate and 50 μL 7% NaOH, incubated for 15 min at room temperature and centrifuged at 3000 rpm for 10 s. Subsequently 150 μL 0.1% DMPD in 5 M HCl and 150 μL 10 mM FeCl_3_ in 1 M HCl were added and vortexed until the pellet was resuspended. The solution was further incubated at room temperature for 20 min, resulting in an absorption wavelength of 670 nm. The optical density was measured with an Infinite 200 plate reader (TECAN) at room temperature. The quantification of iron or sulfur contents was calculated based on calibration curves obtained by measuring standards with an iron or sulfur content of 2–20 nmol.

### EPR spectroscopy

EPR spectra were recorded with an X-band ESP 300 spectrometer (Bruker). Samples were loaded in quartz EPR tubes (707SQ, 250 mm) under strictly anaerobic conditions and frozen in liquid nitrogen. To obtain reduced protein, 10 mM sodium dithionite was added under strict anaerobic condition. The EPR spectra were recorded at 10 K, with a microwave power of 28 dB and 0.47 mW at highest signal intensity. The shown spectra are an average of 16 scans each, with a total scan time of 22 min per spectrum.

### Mössbauer spectroscopy

^57^Fe-labeled ftFliB was produced and purified as described above with supplement of 15–25 μM ammonium ^57^iron citrate instead of metal mix in the bacterial culture. Ammonium ^57^iron citrate was also used for Fe-S cluster reconstitution of ftFliB. Zero-field ^57^Fe Mössbauer measurements on ftFliB (~50 mg) were conducted on a commercial *Halder* and *WissEl* transmission spectrometer with sinusoidal velocity sweep. For the low-temperature measurement at T = 100 K we used a *CryoVac* continuous-flow cryostat with N_2_ exchange gas adjusted at ca. 100 mbar. The temperature was measured with a calibrated silicon diode located close to the sample container, providing a temperature stability of better than 0.1 K. The nominal activity of the ^57^Fe Mössbauer source was 50 mCi of ^57^Co in a rhodium matrix; the source was stored at ambient temperatures during the measurement. Velocity calibration was done with an α-iron foil at ambient temperature; the minimum experimental line width (FWHM) was < 0.24 mm s^-1^. The spectra were analysed by least-square fits using doublets of Lorentzian lines utilizing the software package NORMOS [47]. Isomer shifts (δ) were specified relative to metallic iron at room temperature but were not corrected in terms of the second-order Doppler shift.

### *In vitro* methylation assay

Methyltransferase assays were conducted by mixing methylation substrate flagellin (FliC or FljB) with methyltransferase wild-type ftFliB or ftFliB ^C17A C22A C26A C27A^ mutant. The assay was performed under anaerobic environment in reaction buffer (50 mM Tris-HCl, 5 mM MgCl_2_, 4 mM dithiothreitol, pH 8.5) with 10 μM substrate, 6 μM methyltransferase and 1 mM SAM (Sigma-Aldrich). Reactions were incubated at 25°C for 2 h and heat inactivated by boiling in Laemmli sample buffer for 5 minutes. Samples were analyzed using immunoblotting with antibodies to detect methylated lysine (Cayman).

### Continuous methyltransferase assay

The methylation activity was monitored continuously using the commercial methyltransferase fluorometric assay kit (SAMfluoro: SAM Methyltransferase Assay, G-Biosciences). In brief, the assay was performed by mixing 10 μM flagellin, 6 μM reduced ftFliB and 1 mM SAM. The methylation reaction produced SAH, which was rapidly converted to hydrogen peroxide by the enzyme mix containing AdoHcy nucleosidase, adenine deaminase, and xanthine oxidase. The rate of hydrogen peroxide production was measured by ADHP, producing fluorescent compound resorufin (Ex 530–540 nm/ Em 585-595nm). The fluorescence signal was monitored with an Infinite 200 plate reader (TECAN) at 37°C every 30 s for 30 min. SAH was used as a positive control; reaction without ftFliB was regarded as negative control; mixture without enzyme and substrate was used as background control. The quantification of methyltransferase activity was calculated based on calibration curves obtained by measuring resorufin standards with a content of 1.25–10 μM, using the formula ‘Methyltransferase Activity (nmol/min/ml) = Slope of the linear portion of the reaction curve /Slope of Resorufin standard X Sample Dilution’. One unit of Methyltransferase activity is defined as the amount of enzyme that will cause the formation of 1nmol of fluorophore per minute at 37°C.

### HPLC-MS analysis of methylation products

The reaction products from *in vitro* methylation assay were mixed with methanol and centrifuged at 16,000 g for 20 min. The supernatant was dried with a vacuum evaporator and resuspended in ddH_2_O before applying to HPLC-MS. The HPLC-MS analysis was carried out by UPLC (Waters Acquity system, QDa-Detector) and the assay mixtures were separated on a HSST3 column (150 mm x 2.1 mm, 1.8 μm particle size, Thermo Fisher Scientific). The column was equilibrated in 99% solvent A (ddH_2_O + 0.1% formic acid) and 1% solvent B (acetonitrile + 0.1% formic acid). Linear gradients were applied at a flow rate of 0.4 mL/min: 1–60% solvent B for 5 min, followed by a gradient from 60–100% solvent B for 0.4 min, holding with 100% solvent B for 0.5 min. Column temperature was performed at 45°C.

Detection of substrates and products was performed using electrospray ionization in positive mode (ESI+). The extracted ion chromatograms (EIC) of SAM (m/z 399 [M + H]^+^), SAH (m/z 385 [M + H]^+^), 5’-dA (m/z 252 [M + H]^+^), MTA (m/z 298 [M + H]^+^) were further analyzed using EMPOWER 3 (Waters) software.

### SEC-MALS measurement

Purified protein was loaded onto a TSK-GEL BioAssist G4SWXL column equilibrated with buffer A and eluted at 0.5 mL/min. MALS-RI-UV measurements was performed using a Wyatt mini dawn TREOS 3-angle laser (Wyatt Technology) light scattering coupled to a Wyatt T-rEX RI refractometer. The differential RI increment was set at 0.185 mL/g (25°C at 659 nm). UV spectroscopy was performed on a variable wavelength UV spectrometer (Agilent) at 280 nm. Data analysis was done using ASTRA 7.0.1.24 (Wyatt Technologies).

### AFM imaging

Purified protein was loaded onto a circular MICA slide (Muscovite MICA, Electron Microscopy Sciences), incubated for 15 min and imaged in a droplet of 50 μl or 100 μl. For high-resolution images (512 pt x 512 pt), specimens were examined with a Cypher ES atomic force microscope (Asylum Research), using BL-AC40TS cantilevers (typical spring constant: k ~ 0.1 N·m^−1^, resonant frequency: ω_0_ ~ 110 kHz, Olympus) and a 10°C cooled perfusion cell. Medium resolution images (128 pt x 128 pt) serving as reproductions were taken with a MFP-3D atomic force microscope (Asylum Research) equipped with RC800 PSA cantilevers (constant: k ~ 0.4 N·m^−1^, resonant frequency: ω_0_ ~ 70 kHz, Olympus) at room temperature. The high-resolution images were processed with a median filter (5 x 5 window, 1 pass) using the software MFP-3D under IGOR Pro. Three individual measurements were performed, and 500 nm x 500 nm images were taken at 30 different sample positions.

To determine the mean height of the particles, three 500 nm x 500 nm images from different sample positions of the high-resolution measurement were evaluated with histograms. The fitting with two gauss profiles resulted in an expected value for the ground level and the peak level of the particles. The mean height of all image points allocated to the oligomers was the two expected values. To determine the mean diameter of the particles, four independent section curves were generated in each of the three 500 nm x 500 nm images. The cursor function was used to measure the diameters of all particles that were cut almost centrally, resulting in a total of 112 values.

### TEM imaging

Purified protein was incubated on a carbon-coated copper grid (Electron Microscopy Sciences) at room temperature for 5 minutes and negatively stained with 1% uranyl acetate. Grids were examined using the transmission electron microscope Talos L120C (FEI Thermo Fisher Scientific) at 120 kV. The particle sizes were measured using Fiji [43] with a total number of 100 from four sample preparations.

### Alignment and lysine methylation sites mapping

The reconstitution maps for full length R- type straight FliC (PDB ID: 1UCU) flagellin and flagellar assembly from *S*. *enterica* were kindly provided by Keichi Namba [10]. Lysine methylation sites were highlighted using the molecular graphics PyMOL 2.2.3 (Schrödinger, LLC.) based on previous reports [14,15]. Protein sequence alignments were generated with the server ESPript [48].

### Thermal unfolding, nanoDSF

Fluorescence based thermal experiments were performed using Prometheus NT.48 device (NanoTemper Technologies). Capillaries containing 10 μL purified proteins (~0.5 mg/mL) were loaded; sample buffer was used as a control. The temperature was increased by a rate of 1°C/min from 15°C to 75°C and the fluorescence at emission wavelengths of 330 nm and 350 nm was measured. Backreflection optics monitoring aggregation induced scattering of light by particles was conducted in parallel. For interpretation of spectra, the PR.Control software was applied.

### X-ray fluorescence (XRF) and X-ray absorption near edge structure (XANES)

The iron XRF and K-edge XANES measurements were carried out using the setup at the beamline P11 at PETRA III, DESY, Hamburg [49]. Purified ftFliB was concentrated to around 0.3 mM and loaded onto a nylon loop and shot with x-ray beam at 10.2 keV for emission spectra and starting from 7.04 keV for the absorption edge scan. For the measurement, flat beam with a 200 μm pinhole and 1% were applied. The acquisition time was conducted for 1 s with a Vortex fluorescence detector. The speed of the scan for the XANES was 1 eV/s.

### Purification of flagellin

The flagellin genes *fliC* or *fljB* amplified from *S*. *enterica* serovar Typhimurium strain SL1344 were cloned into vector pASK-IBA3plus (IBA GmbH), respectively. *E*. *coli* BL21 (DE3) carrying these constructs were grown at 37°C in LB supplemented with 100 μg/mL ampicillin. Target gene expression was induced at an OD_600_ of 0.5 by addition of 200 ng/mL anhydrotetracycline hydrochloride (AHT) for 3 h and cells were collected with centrifugation. The cell pellets were resuspended in buffer A supplemented with 2 mg/mL lysozyme, complete EDTA-free protease inhibitor cocktail and 10 μg/ml DNase I. Cell lysis was achieved by sonication, and debris was removed by centrifugation at 48,000 g for 45 min. The flagellin proteins were purified by Strep-Tactin affinity chromatography and eluted with buffer A supplemented with 7.5 mM desthiobiotin. Affinity-purified proteins were polished by SEC on Superdex 200 column (GE Healthcare) equilibrated with buffer A. Peak fractions containing FliC or FljB were pooled and analyzed by SDS-PAGE followed by Coomassie-staining or Western blot.

### Microscale Thermophoresis (MST)

MST experiments were performed using the Monolith NT.LabelFree (NanoTemper Technologies GmbH). MST measurements were done according to the manufacturer’s instructions. In brief, a constant protein concentration of 1 μM of purified ftFliB diluted in assay buffer (50 mM Tris-HCl, 150 mM NaCl, 10% glycerol, 0.1% Pluronic F-127, pH 8.5) was used. To this, a serial dilution of SAM dissolved in assay buffer was added. After a short incubation of 5 min, samples were filled into NT. LabelFree Zero Background MST Premium coated capillaries (NanoTemper Technologies GmbH). Measurements were carried out at 25°C. MST traces were collected with an LED excitation power of 20% and an MST laser power of 20%. For analyzing the interaction affinity, the dissociation constant Kd was calculated using the NanoTemper MO.Affinity Analysis software. Changes in the normalized fluorescence (ΔFnorm [‰]) were plotted as fraction bound.

## Supporting information

S1 Fig Flagellin and flagellar methylation pattern. (**A**) Cartoon and surface representation of full length FliC protomer (PDB ID: 1UCU) [10]. The flagellin domains are indicated, the FliB dependent methylated lysine residues are highlighted in orange based on previous reports [14,15], unmethylated lysine residues are shown in cyan. (**B**) Side view and top view of a flagellar filament assembly of 22 flagellin subunits (PDB ID: 1UCU). Methylated lysine residues are highlighted in orange.
(TIF)

S2 Fig Alignments of flagellin N-lysine methyltransferase FliB from multiple genera. The strains representing the different genera are *S*. *enterica* (WP_00659236), *C*. *amalonaticus* (WP_103774934), *F*. *pulveris* (WP_029591973), *Leclercia* MULTISPECIES (WP_103822438), *E*. *cloacae* (WP_063622014), *Pantoea* sp. IMH (WP_024964901), *Y*. *enterocolitica* (WP_077175448), *A*. *caviae* (WP_113979125), *Shewanella* multispecies (WP_101090854) and *Brevibacillus* sp. NRRL NRS-1210 (WP_106838065), respectively.
(TIF)

S3 Fig Alignments of D0 domain truncated flagellins from multiple genera. The stains representing the genera are *S*. *enterica* FliC (WP_000079805) and FljB (WP_000079794), *Y*. *enterocolitica* (WP_133155043), *E*. *coli* (WP_061353988), *C*. *sedlakii* (WP_042284015), and *E*. *cloacae* (WP_063940424), respectively. The domains are indicated above the alignment. FliB dependent methylated lysine residues of *S*. *enterica* FliC are indicated with stars based on previous reports [14,15]. The sequence alignments were conducted using AliView [50] and analyzed using ESPript 3.0 [48], with the color codes “Red box, white character” means as “Strict identity”; “Red character or black bold” as “Similarity in a group”; “Blue frame” as “Similarity across groups”.
(TIF)

S4 Fig Confocal microscopy images of FliB stained *Salmonella* cells. (**A**) High magnification micrographs visualizing the FliB localization in *Salmonella* cells. FliB was detected by using an anti-strep primary antibody in combination with a secondary antibody conjugated with the Alexa 647 (red) and the cell membrane was labelled with *FAST* DiO lipid dye (green). Scale bar is 1 μm. (**B**) Overview of FliB stained *Salmonella* cells. Scale bar is 10 μm. (**C**) Overview of control *Salmonella* cells with staining lacking anti-strep primary antibody for detection of FliB. Scale bar is 10 μm.
(TIF)

S5 Fig FliB construct and purification. (**A**) Fusion protein with N-terminal His_6_ tag, truncated FleB (FleBt) solubility tag containing residues 54 to 332 and FliB at C terminal, protein residue numbers are listed above. (**B**) Coomassie stained SDS-PAGE (left) and Western blot (right) of His-tagged ftFliB fusion protein purified from *E*. *coli*. Molecular weight markers (M) are indicated.
(TIF)

S6 Fig Stability analysis of ftFliB. (**A**) Analysis of ftFliB stability by nanoDSF in Buffer A (50 mM Tris-HCl, 150 mM NaCl, 10% glycerol, pH 8.5). Capillaries containing 10 μL purified proteins (~0.5 mg/mL) were measured at fluorescence emission wavelengths of 330 nm and 350 nm, with a temperature range from 15°C to 75°C increased by a rate of 1°C/min. (**B**) Analysis of ftFliB stability by nanoDSF with aggregation scattering detection in buffer A.
(TIF)

S7 Fig FRET spectroscopy of liposomes with buffer or FleBt tag. PE:PG (1:1 [m:m]) liposomes were double labelled with NBD-PE and rh-PE and had a total lipid concentration of 10 μM. Solubility tag FleBt molar concentrations and buffer volume was adapted to the ftFliB experiments (Fig 1C). At 22°C, neither FleBt nor buffer titration lead to detectable signal changes at pH values 8.5 and 6.5. At 37°C only minor signal changes occurred for FleBt and buffer at pH values 8.5 and 6.5. Error bars indicate standard deviations of 4 or 5 independent measurements.
(TIF)

S8 Fig Characterization of FliB [4Fe-4S] cluster. (**A**) Protein color of affinity purified ftFliB. ftFliB showed a light brown color, suggesting an incomplete load of iron-sulfur clusters. (**B**) Protein color of reconstituted ftFliB. After reconstitution with additional iron and sulfide under anaerobic environment, protein showed a dark brown color. (**C**) Protein color of reduced ftFliB. The reconstituted protein was incubation with 10 mM dithionite overnight, reduced ftFliB was colorless. (**D**) X-ray fluorescence (XRF) spectrum of ftFliB protein sample (~ 0.3 mM). An x-ray beam of 10.2 keV was shot on the protein solution (~0.3 mM), iron Kα (~6.4 keV) and Kβ (~7.0 keV) peaks were detected. Sample buffer was used as a background control. (**E**) Iron K-edge XANES spectrum of ftFliB protein sample. An X-ray energy scan from 7.04–7.16 keV was shot on the protein solution (~0.3 mM), XANES shift corresponding to the theoretical absorption energy of iron (K-edge 7.1120 keV) was detected. Buffer was used as a background control.
(TIF)
