# Supplementary figures and images for: Flagellin lysine methyltransferase FliB catalyzes a [4Fe-4S] mediated methyl transfer reaction
Source: PLoS Pathog. 2021 Nov 17;17(11):e1010052. doi: 10.1371/journal.ppat.1010052 (PMC8598068; doi:10.1371/journal.ppat.1010052)

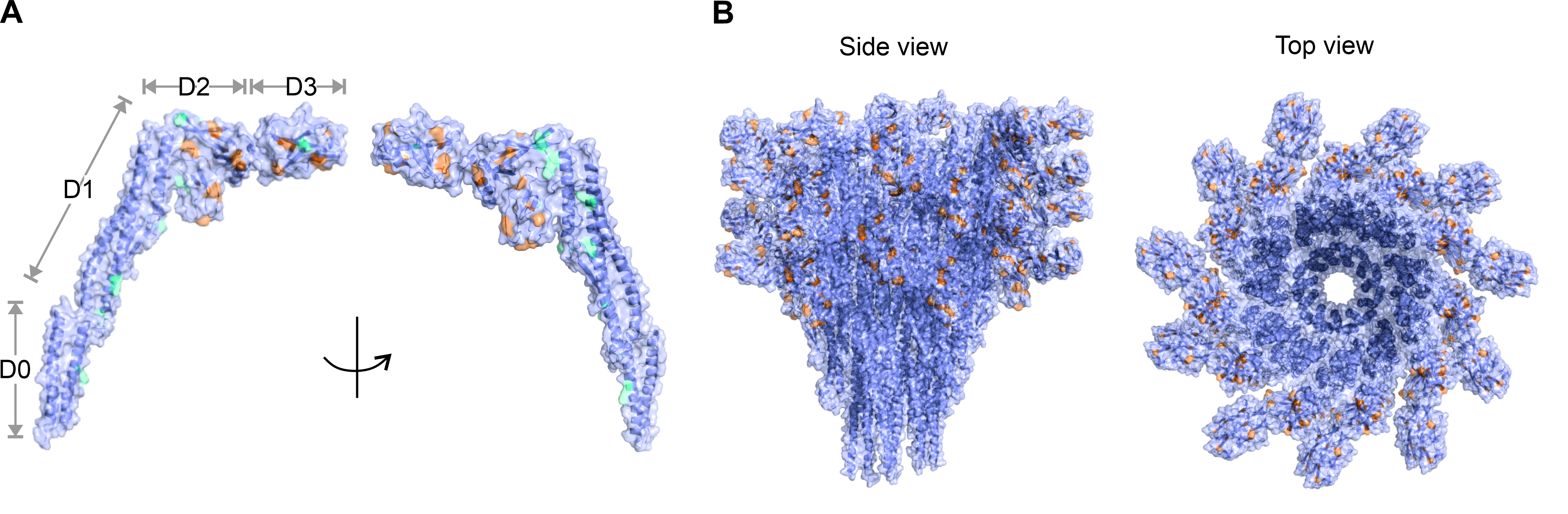

Supplement: S1 Fig — (A) Cartoon and surface representation of full length FliC protomer (PDB ID: 1UCU) [10]. The flagellin domains are indicated, the FliB dependent methylated lysine residues are highlighted in orange based on previous reports [14,15], unmethylated lysine residues are shown in cyan. (B) Side view and top view of a flagellar filament assembly of 22 flagellin subunits (PDB ID: 1UCU). Methylated lysine residues are highlighted in orange. (TIF) [file ppat.1010052.s001.tif]

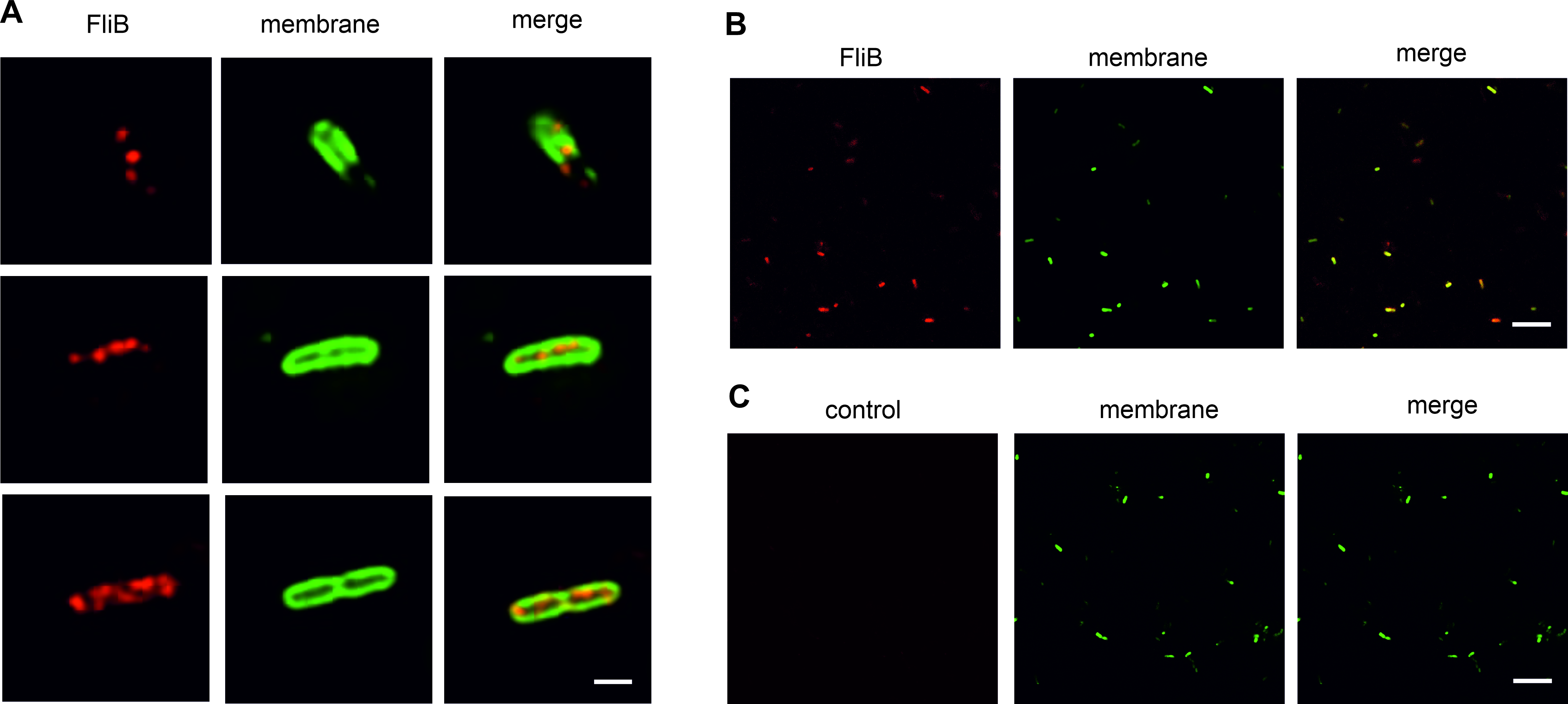

Supplement: S4 Fig — (A) High magnification micrographs visualizing the FliB localization in Salmonella cells. FliB was detected by using an anti-strep primary antibody in combination with a secondary antibody conjugated with the Alexa 647 (red) and the cell membrane was labelled with FAST DiO lipid dye (green). Scale bar is 1 μm. (B) Overview of FliB stained Salmonella cells. Scale bar is 10 μm. (C) Overview of control Salmonella cells with staining lacking anti-strep primary antibody for detection of FliB. Scale bar is 10 μm. (TIF) [file ppat.1010052.s004.tif]

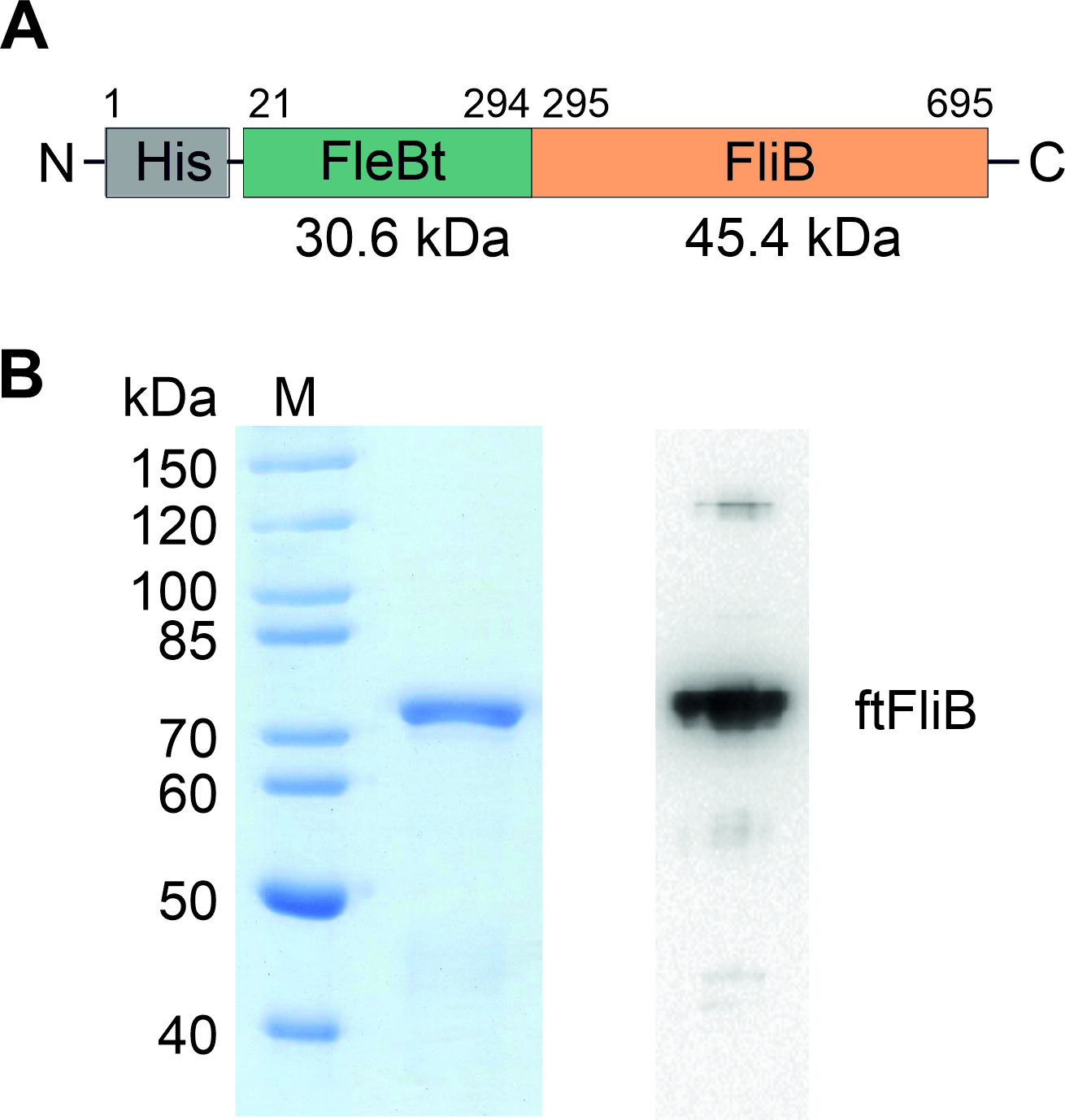

Supplement: S5 Fig — (A) Fusion protein with N-terminal His6 tag, truncated FleB (FleBt) solubility tag containing residues 54 to 332 and FliB at C terminal, protein residue numbers are listed above. (B) Coomassie stained SDS-PAGE (left) and Western blot (right) of His-tagged ftFliB fusion protein purified from E. coli. Molecular weight markers (M) are indicated. (TIF) [file ppat.1010052.s005.tif]

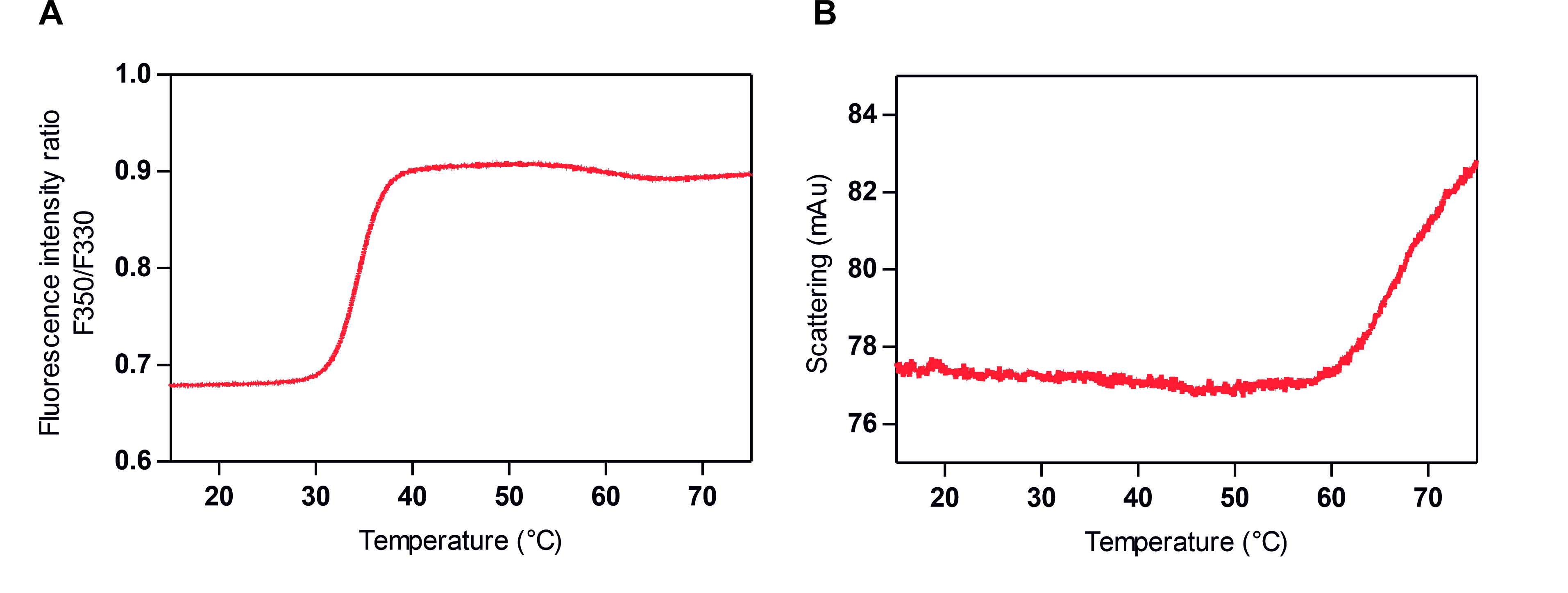

Supplement: S6 Fig — (A) Analysis of ftFliB stability by nanoDSF in Buffer A (50 mM Tris-HCl, 150 mM NaCl, 10% glycerol, pH 8.5). Capillaries containing 10 μL purified proteins (~0.5 mg/mL) were measured at fluorescence emission wavelengths of 330 nm and 350 nm, with a temperature range from 15°C to 75°C increased by a rate of 1°C/min. (B) Analysis of ftFliB stability by nanoDSF with aggregation scattering detection in buffer A. (TIF) [file ppat.1010052.s006.tif]

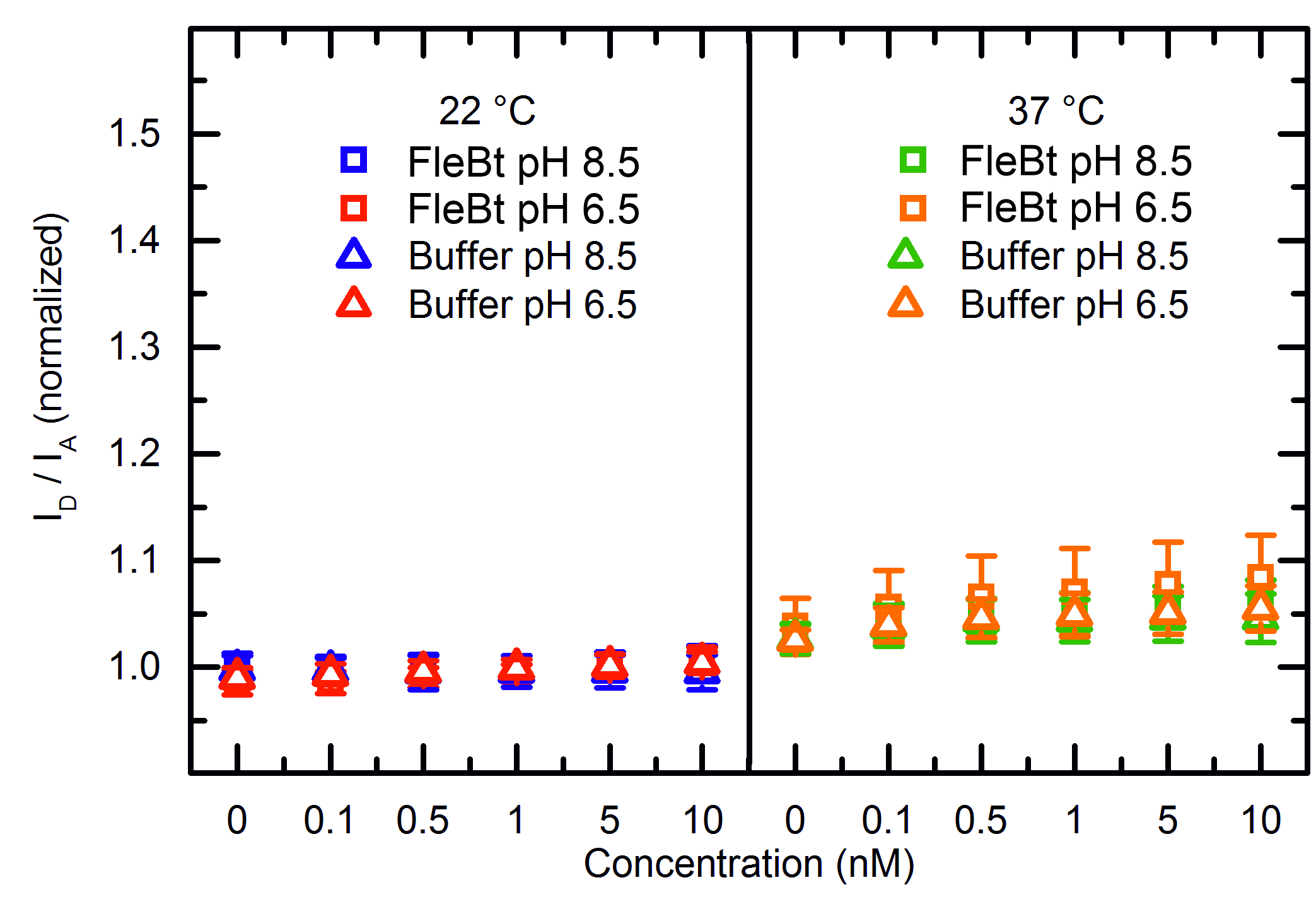

Supplement: S7 Fig — PE:PG (1:1 [m:m]) liposomes were double labelled with NBD-PE and rh-PE and had a total lipid concentration of 10 μM. Solubility tag FleBt molar concentrations and buffer volume was adapted to the ftFliB experiments (Fig 1C). At 22°C, neither FleBt nor buffer titration lead to detectable signal changes at pH values 8.5 and 6.5. At 37°C only minor signal changes occurred for FleBt and buffer at pH values 8.5 and 6.5. Error bars indicate standard deviations of 4 or 5 independent measurements. (TIF) [file ppat.1010052.s007.tif]

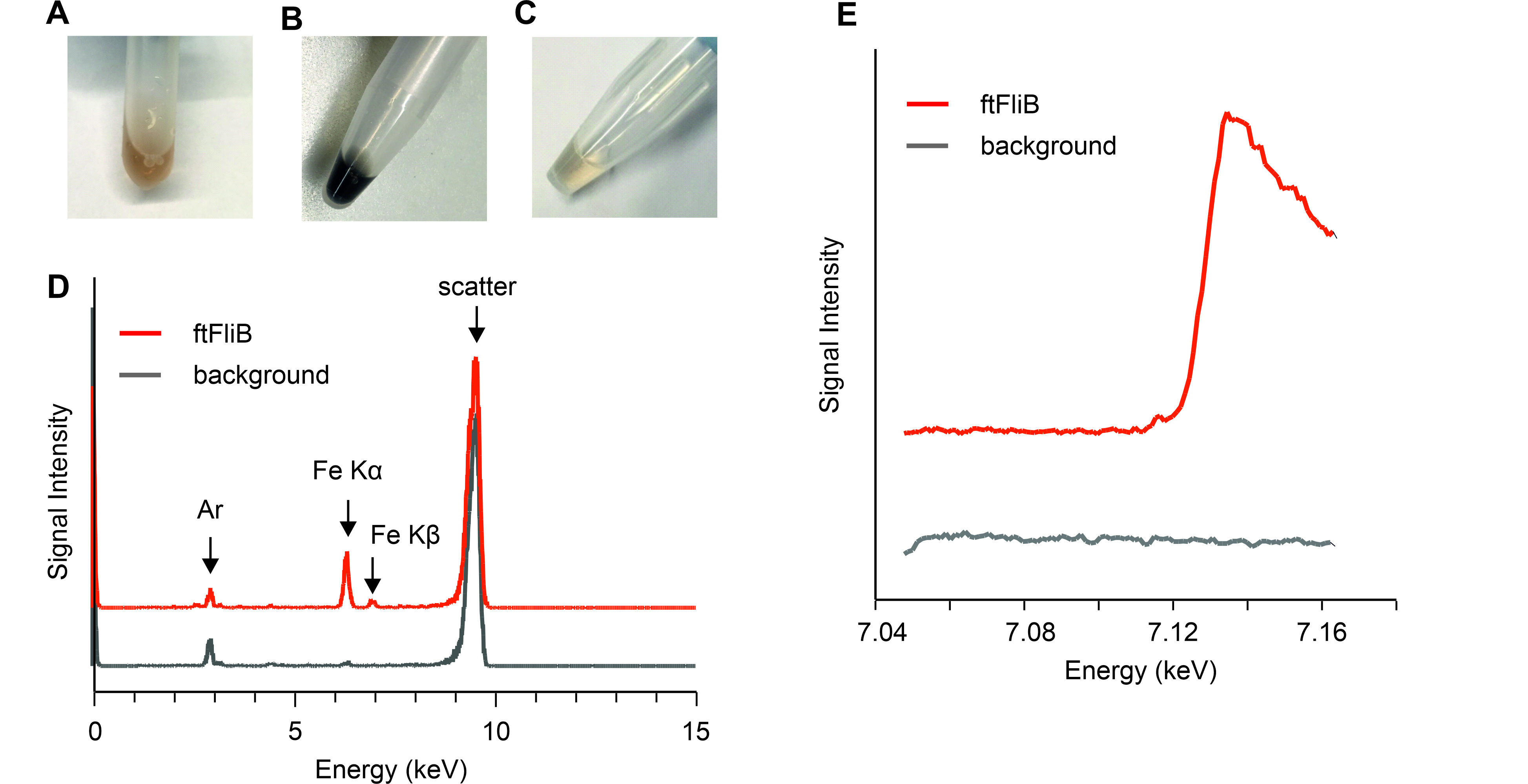

Supplement: S8 Fig — (A) Protein color of affinity purified ftFliB. ftFliB showed a light brown color, suggesting an incomplete load of iron-sulfur clusters. (B) Protein color of reconstituted ftFliB. After reconstitution with additional iron and sulfide under anaerobic environment, protein showed a dark brown color. (C) Protein color of reduced ftFliB. The reconstituted protein was incubation with 10 mM dithionite overnight, reduced ftFliB was colorless. (D) X-ray fluorescence (XRF) spectrum of ftFliB protein sample (~ 0.3 mM). An x-ray beam of 10.2 keV was shot on the protein solution (~0.3 mM), iron Kα (~6.4 keV) and Kβ (~7.0 keV) peaks were detected. Sample buffer was used as a background control. (E) Iron K-edge XANES spectrum of ftFliB protein sample. An X-ray energy scan from 7.04–7.16 keV was shot on the protein solution (~0.3 mM), XANES shift corresponding to the theoretical absorption energy of iron (K-edge 7.1120 keV) was detected. Buffer was used as a background control. (TIF) [file ppat.1010052.s008.tif]
